# Supplementary material for: The interactome of intact mitochondria by cross-linking mass spectrometry provides evidence for coexisting respiratory supercomplexes
Source: Mol Cell Proteomics. 2017 Dec 8;17(2):216–32. doi: 10.1074/mcp.RA117.000470 (PMC5795388; doi:10.1074/mcp.RA117.000470)
Supplement: Supplemental Data [file supp_RA117.000470_133922_0_supp_23978_fzffwf.zip › spectra_annotation/mito_DR_spectra_annotation/134-1-14-1-5-1.pdf]

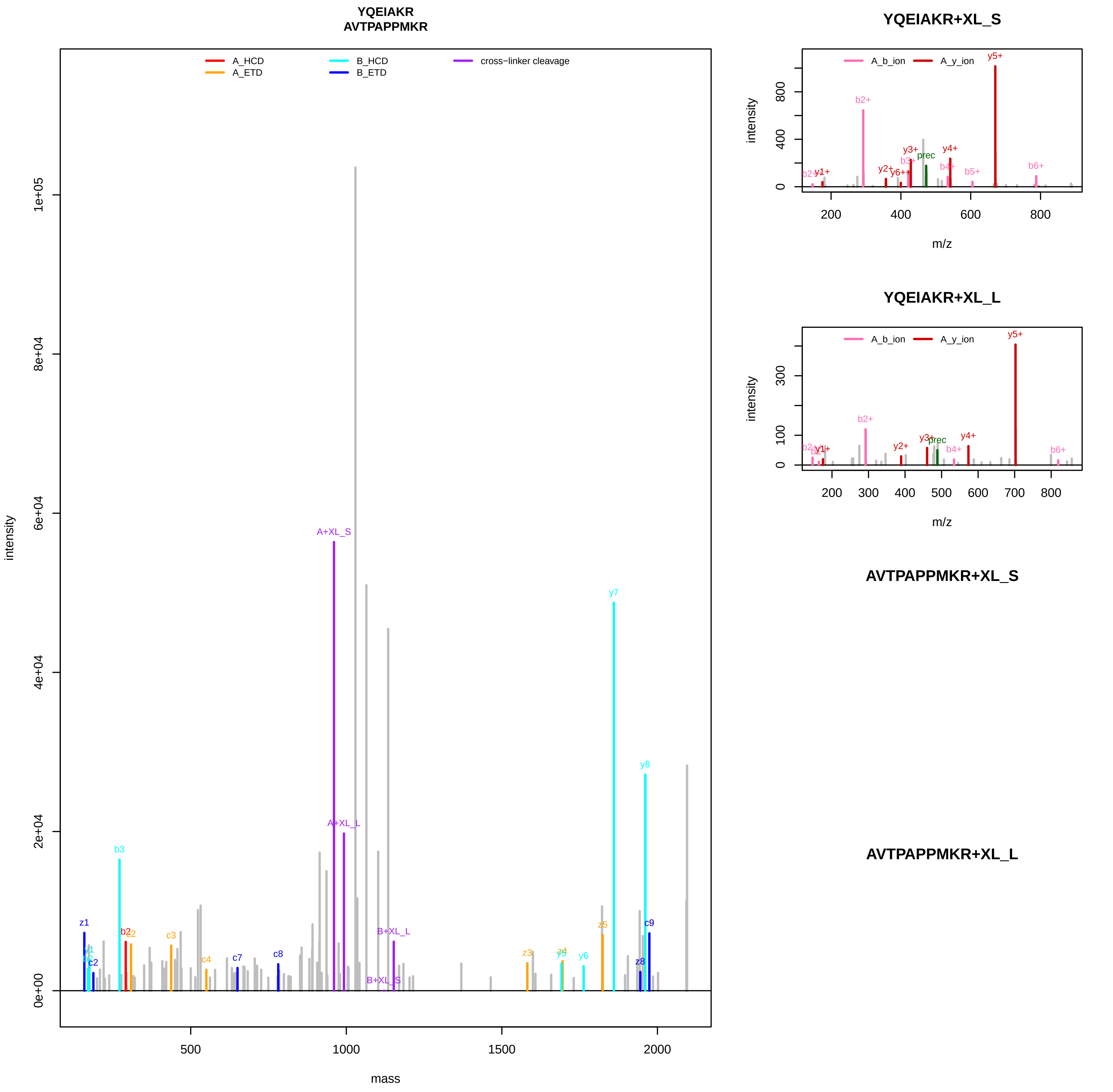
YQEIAKR
AVTPAPPMKR
A_HCD
A_ETD
B_HCD
B_ETD
cross−linker cleavage
intensity
mass
0e+00
2e+04
4e+04
6e+04
8e+04
1e+05
500
1000
1500
2000
A+XL_S
A+XL_L
B+XL_L
B+XL_S
y7
y8
b3
z1
b2
c2
c3
c4
c7
c8
c9
z3
z4
z5
z8
y5
y6
YQEIAKR+XL_S
A_b_ion
A_y_ion
y5+
b2+
y3+
y4+
prec
b6+
b5+
b4+
b3+
y2+
y6++
y1+
m/z
YQEIAKR+XL_L
AVTPAPPMKR+XL_S
AVTPAPPMKR+XL_L
